# Supplementary material for: Application Prospects of FTIR Spectroscopy and CLSM to Monitor the Drugs Interaction with Bacteria Cells Localized in Macrophages for Diagnosis and Treatment Control of Respiratory Diseases
Source: Diagnostics (Basel). 2023 Feb 12;13(4):698. doi: 10.3390/diagnostics13040698 (PMC9954918; doi:10.3390/diagnostics13040698)

Supplementary Materials

Application prospects of FTIR spectroscopy and CLSM to monitor the drugs interaction with bacteria cells localized in macrophages for diagnosis and treatment control of respiratory diseases

Igor D. Zlotnikov [1], Alexander A. Ezhov [2], Maksim A. Vigovskiy [3,4], Olga A. Grigorieva [3,4], Uliana D. Dyachkova [3,4], Natalya G. Belogurova [1], and Elena V. Kudryashova [1,*]

1 Faculty of Chemistry, Lomonosov Moscow State University, Leninskie Gory, 1/3, 119991 Moscow, Russia

2 Faculty of Physics, Lomonosov Moscow State University, Leninskie Gory, 1/2, 119991 Moscow, Russia

3 Medical Research and Education Center, Institute for Regenerative Medicine, Lomonosov Moscow State University, 27/10, Lomonosovsky Ave., 119192 Moscow, Russia

4 Faculty of Medicine, Lomonosov Moscow State University, 27/1, Lomonosovsky prosp., 119192 Moscow, Russia

* Correspondence: Helena_koudriachova@hotmail.com (E.V.K.)

Content

Figure S1. FTIR spectra of *E. coli* incubated with drug delivery system HPCD-PEI1.8-triMan. T = 22 °C.

Figure S2. Emission fluorescence spectra of FITC (drug delivery system labelled), eosin (*E. coli* labelled) and Dox obtaining by CLSM. λ_{em} = 488 nm (multiline Argon laser).

Figure S3. Confocal laser scanning images of CD206+ macrophages with absorbed eosin-labelled E. coli. Incubation with Dox and FITC-labeled HPCD-PEI1.8-triMan. The scale segment is 100 µm (division value is 20 µm); 4–6 channels are shown: red, Dox; green, FITC; magenta, eosin; gray, transmission light mode; and overlay. λ_{em} = 488 nm (multiline Argon laser).

Figure S1. FTIR spectra of *E. coli* incubated with drug delivery system HPCD-PEI1.8-triMan. T = 22 °C.

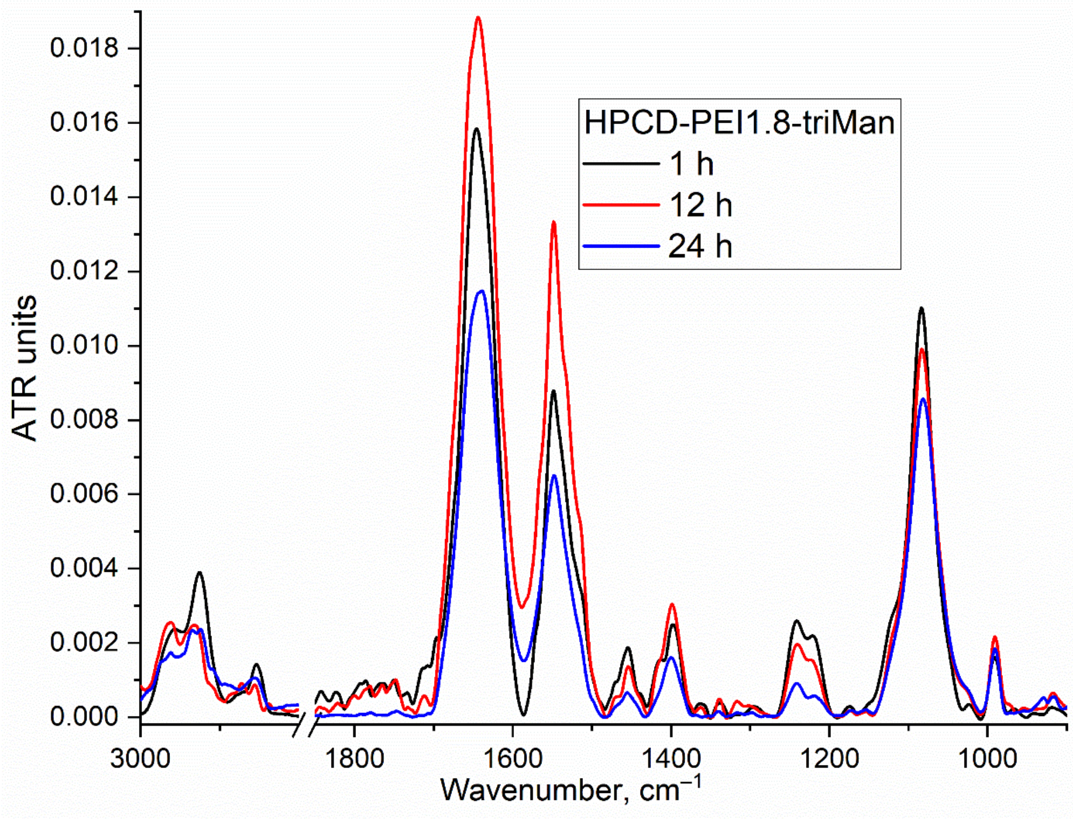

Figure S2. Emission fluorescence spectra of FITC (drug delivery system labelled), eosin (*E. coli* labelled) and Dox obtaining by CLSM. λ_{em} = 488 nm (multiline Argon laser).

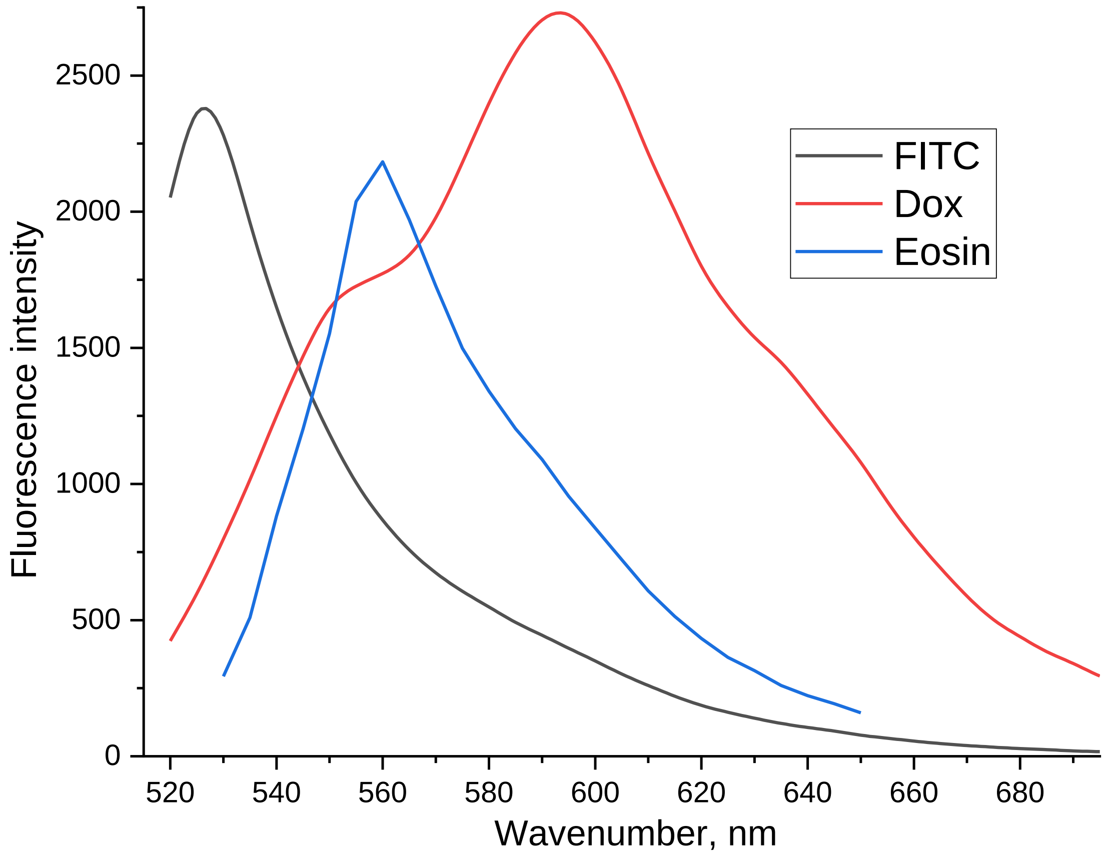

Figure S3. Confocal laser scanning images of CD206+ macrophages with absorbed eo-sin-labelled E. coli. Incubation with Dox and FITC-labeled HPCD-PEI1.8-triMan. The scale segment is 100 μm (division value is 20 μm); 4–6 channels are shown: red, Dox; green, FITC; magenta, eosin; gray, transmission light mode; and overlay. λ_{em} = 488 nm (multiline Argon laser).

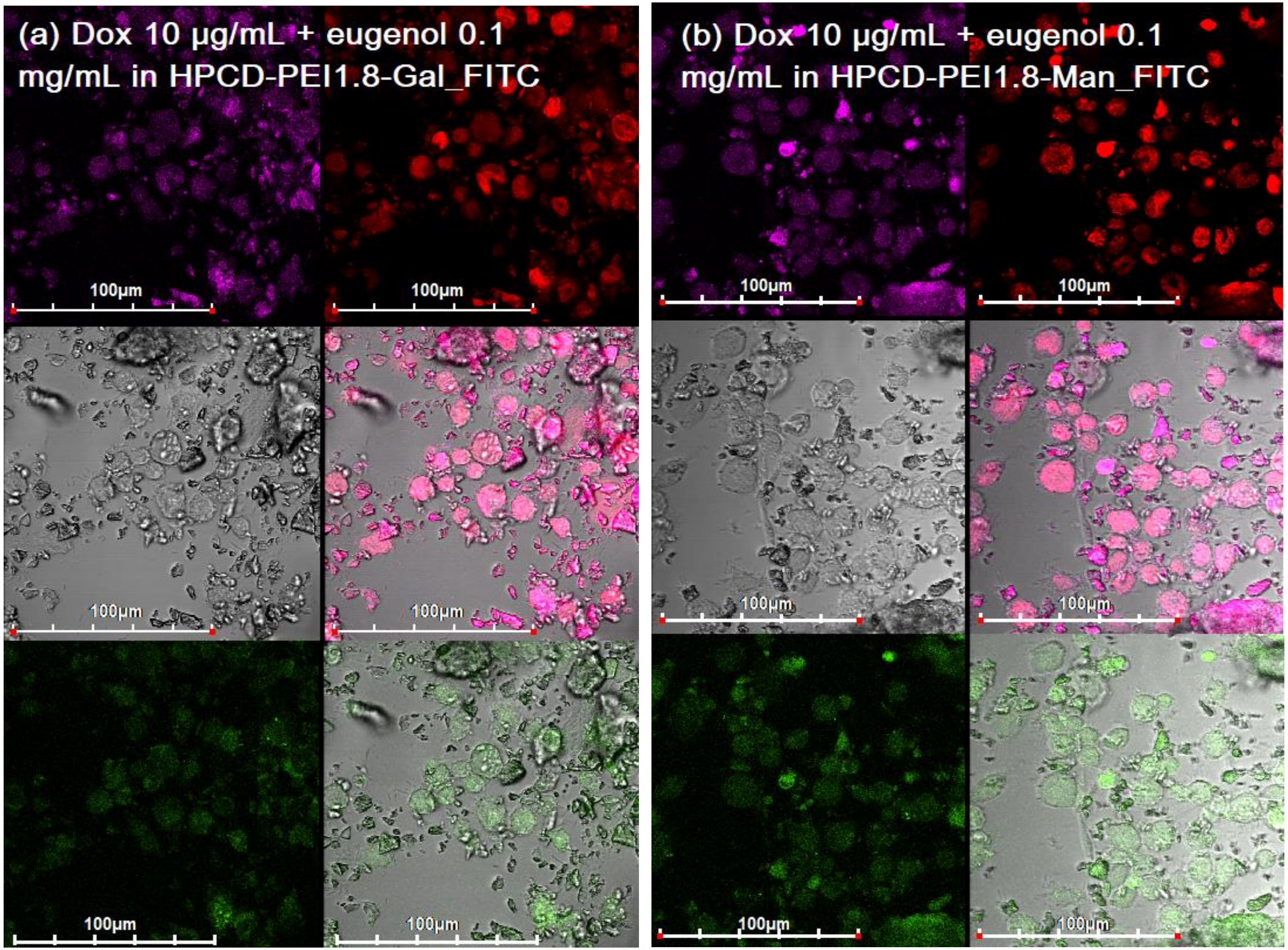

Supplement: Supplementary file 1 [file diagnostics-13-00698-s001.zip › diagnostics-2153349-supplementary.pdf]
